# Regorafenib combined with irinotecan as second-line treatment in metastatic gastro-oesophageal adenocarcinomas: results of PRODIGE 58–UCGI35–REGIRI Unicancer randomised phase II study

**DOI:** 10.1016/j.esmoop.2025.105096

**Published:** 2025-05-12

**Authors:** E. Samalin, L. Evesque, A. Turpin, C. De La Fouchardiere, F. Khemissa-Akouz, O. Bouché, M. Muller, S. Dermeche, D. Botsen, D. Tougeron, A. Zaanan, M. Ben Abdelghani, E. Guardiola, O. Dubreuil, V. Le Brun Ly, A. Hennequin, S. Watson, D. Sefrioui, T. Lecomte, N. De Sousa Carvalho, A. Hulin, E. Crapez, F. Castan, H. Senellart

**Affiliations:** 1 ICM, Department of Medical Oncology, Université de Montpellier, Montpellier, France; 2 Centre Antoine Lacassagne, Nice, France; 3 Department of Medical Oncology, CHRU Lille, Lille, France; 4 CNRS INSERM UMR9020-U1277, CANTHER Cancer Heterogeneity Plasticity and Resistance to Therapies, Université de Lille, Lille, France; 5 Centre Léon Bérard, Lyon, France; 6 Hôpital Saint Jean, Perpignan, France; 7 CHU Reims, Reims, France; 8 CHRU de Nancy, Vandoeuvre-Les-Nancy, France; 9 Institut Paoli Calmettes, Marseille, France; 10 Department of Medical Oncology, Institut Godinot, Reims, France; 11 CHU Poitiers, Poitiers, France; 12 Hôpital Européen George Pompidou, Paris, France; 13 Centre Paul Strauss, Strasbourg, France; 14 Centre de Cancérologie du Grand Montpellier, Montpellier, France; 15 GH Diaconesses, Paris, France; 16 CHU Limoges—Hôpital Dupuytren, Limoges, France; 17 Centre Georges François Leclerc, Dijon, France; 18 Institut Curie, Paris, France; 19 CHU Rouen, Rouen, France; 20 CHRU Tours—Hôpital Trousseau, Chambray-lès-Tours, France; 21 Unicancer—UCGI Group, Paris, France; 22 APHP, GH H Mondor, Créteil, France; 23 ICM, Translational Research Unit, Montpellier, France; 24 ICO René Gauducheau, Saint Herblain, France

**Keywords:** regorafenib (REGO), irinotecan (IRI), metastatic gastro-oesophageal adenocarcinomas (mGA)

## Abstract

**Background:**

Several options have been evaluated in metastatic gastro-oesophageal adenocarcinomas (mGA) after failure of first-line fluoropyrimidine and platinum-based chemotherapy. Regorafenib (REGO), a receptor tyrosine kinase inhibitor, has shown promising activity as second- and third-line treatment of mGA.

**Patients and methods:**

PRODIGE58-UCGI35-REGIRI was a comparative, prospective, phase II, open-label study evaluating the safety and efficacy of REGO [160 mg/day on day 2 (D2)-D8/D16-D22] plus irinotecan (IRI: 180 mg/m^2^ intravenously on D1/D15 every 28 days) versus IRI alone in patients with mGA (gastric or gastro-oesophageal junction/tumour Siewert II and III) after failure of first-line fluoropyrimidine and platinum-based chemotherapy. Primary endpoint was overall survival (OS).

**Results:**

Forty-four patients were included in the REGIRI arm and 45 in the IRI arm, primary tumours (67.4%) were mainly localised in the gastro-oesophageal junction, and 60.7% patients had synchronous metastases. With a median follow-up of 19.4 months [95% confidence interval (CI) 16.8-29.9 months], median OS was 6.3 months (95% CI 5.2-7.1 months) versus 8.2 months (95% CI 5.2-9.7 months) in the REGIRI versus IRI arms (hazard ratio 1.11, 95% CI 0.70-1.74, *P* = 0.66). Median progression-free survival was 2.2 months versus 1.9 months, objective response rate 15.9% versus 13.3%, and disease control rate 45.5% versus 33.3%. Grade 3 treatment-related adverse events (AEs) were reported for 52.3% of patients in the REGIRI arm versus 23.3% in the IRI arm with four toxic deaths (two homozygous *UGT1A1∗28* patients died from sepsis and thrombotic microangiopathy, and two heterozygous *UGT1A1∗1/∗28* patients from diarrhoea and pulmonary embolism), versus one (*UGT1A1∗1* wild-type patient died from primary tumour perforation). Main grade ≥3 AEs were diarrhoea (18.2% versus 7.0%), hypertension (9.1% versus 0.0%), asthenia (6.8% versus 0.0%), febrile neutropenia (6.8% versus 0.0%), neutropenia (6.8% versus 11.6%), and weight decrease (6.8% versus 0.0%).

**Conclusions:**

The study was stopped early because of limited efficacy and increased toxicities in the REGIRI arm, possibly due to drug interactions. No optimal sub-population that could benefit from a REGIRI regimen exposure was identified.

## Highlights

• The REGIRI study showed severe toxicities in the REGIRI arm possibly due to drug interactions.
• Pre-therapeutic UGT1A1 variants analysis and IRI progressive increase could help manage REGIRI-induced toxicities.
• Gastrectomy was identified as a potential prognostic factor for OS.

## Introduction

Gastric cancer (GC) accounts for 5.6% of all cancers and in 2020, >1 million new cases were diagnosed worldwide. It ranks fifth among the most common cancers after breast, lung, colorectal, and prostate, and with around 768 000 subsequent deaths, represents the fourth leading cause of cancer-related death.^1^ GC incidence and mortality are highly variable depending on region (highest rates are observed in Eastern Asia, Central and Eastern Europe, and South America), diet, and *Helicobacter pylori* infection.^2^, ^3^, ^4^ Survival rates have steadily improved over the past few decades, thanks to earlier detection and better treatment options, but advanced GC remains a serious global health burden.^5^ Surgical resection of GCs, especially in the early stages, is potentially curative. Most patients, however, still experience disease relapse after resection, and ∼50% of them present with non-operable or metastatic disease at diagnosis.^6^

Fluoropyrimidine and platinum-based chemotherapy forms the backbone of first-line (1L) treatment for advanced GCs with a historical median overall survival (OS) of <1 year in non-Asian countries.^7^ The addition of trastuzumab, a human epidermal growth factor receptor 2 (HER2) inhibitor targeted therapy, to chemotherapy has improved survival in patients with HER2-positive advanced GCs, and results showed that the further addition of pembrolizumab, an anti-programmed cell death protein 1 (PD-1) monoclonal antibody, to this combination demonstrated a survival benefit in those with programmed death-ligand 1 (PD-L1)-combined positive score (CPS) ≥1.^8^^,^^9^ In patients with HER2-negative advanced GCs, the combination of chemotherapy and nivolumab, another PD-1 inhibitor, or pembrolizumab, also exhibited a better survival for patients with PD-L1-CPS ≥5 or CPS ≥1, respectively.^10^, ^11^, ^12^ Nivolumab and pembrolizumab received approval from the European Medicines Agency in this indication in 2021 and 2023, respectively. Benefits are limited since up to 30% of patients display progression at first tumour evaluation and median progression-free survival (PFS) is only 4-7 months.^13^^,^^14^ Following 1L failure, ∼50% of patients are medically fit to receive second-line (2L) therapies. The selection of 2L regimen depends on prior therapy and performance status (PS).^15^ Cytotoxic agents that have not already been used in 1L can be attempted. Paclitaxel and docetaxel, as monotherapies, and irinotecan (IRI), as monotherapy or with 5-fluorouracil (5-FU)–leucovorin, i.e. FOLFIRI, are different options with similar efficacy in terms of OS.^16^, ^17^, ^18^, ^19^, ^20^ Recently, immune checkpoint inhibitors combined with FOLFIRI in advanced gastric/oesophagogastric junction adenocarcinoma showed an acceptable safety profile and antitumour activity in a subgroup of patients.^21^ Drugs that target multiple receptor tyrosine kinases involved in angiogenesis have also shown better overall response rate (ORR), PFS, and OS.^22^, ^23^, ^24^, ^25^ Incidentally, ramucirumab, alone or combined with paclitaxel, is now the standard treatment as 2L therapy, but this drug is currently not reimbursed in France. In addition, we also conducted the GASTFOX trial, which investigated the benefits of triplet chemotherapy with the addition of docetaxel to FOLFOX (modified FLOT) as 1L treatment, reported in ESMO in 2023.^26^ Since the positive results of the GASTFOX trial, the use of this combination was preferred in patients HER2-negative, CPS ≤5, and fit to receive triplet regimen. Consistently, results from the phase II INTEGRATE trial demonstrated that regorafenib (REGO), an oral tyrosine kinase inhibitor, improved the clinical outcome of advanced gastro-oesophageal adenocarcinoma (GA) patients refractory to two or more previous lines of chemotherapy.^27^^,^^28^ REGO blocks the activity of kinases involved in tumour angiogenesis [vascular endothelial growth factor receptor 1 (VEGFR1), VEGFR2, VEGFR3, TIE2], oncogenesis (KIT, RET, RAF-1, BRAF), and tumour microenvironment (platelet-derived growth factor receptor, fibroblast growth factor receptor), and might represent an effective 2L therapy for advanced GCs.

The REGIRI (PRODIGE58-UCGI 35) study was conducted to explore the efficacy and safety of REGO combined with IRI compared with IRI monotherapy as 2L treatment of metastatic GA (mGA), after failure of 1L fluoropyrimidine and platinum-based chemotherapy.

## Patients and methods

### Study design

PRODIGE58-UCGI35-REGIRI was a comparative, interventional, prospective, phase II, randomised, open-label, multicentric study. The trial was sponsored by Unicancer and conducted within the PRODIGE group. It was approved by French regulatory authorities (9 November 2018) and French ethics committee CPP Ouest V-Rennes (10 October 2019). All patients provided written informed consent. This study is registered in EudraCT (2018-002374-46) and ClinicalTrials.gov (NCT03722108).

### Patient eligibility

Eligible patients were ≥18 years old with a histologically confirmed diagnosis of gastro-oesophageal junction (Siewert II and III) or gastric adenocarcinomas, and asymptomatic primary tumour (e.g. no dysphagia leading to trouble swallowing tablets, no bleeding requiring repeated blood transfusion), metastatic disease, at least one target lesion according to RECIST v1.1, disease progression after 1L fluoropyrimidine and platinum agent-based chemotherapy or early recurrent disease after surgery with neoadjuvant and/or adjuvant platinum-based chemotherapy (within 6 months of the end of chemotherapy) or progression during neoadjuvant and/or adjuvant platinum-based chemotherapy (5-FU or 5-FU prodrugs combined with cisplatin or oxaliplatin), and Eastern Cooperative Oncology Group (ECOG) PS ≤1. Key exclusion criteria comprised symptomatic brain metastases or carcinomatous meningitis, bone-only metastasis, cardiac, pulmonary, gastrointestinal, hepatobiliary, vascular, or nervous system toxicities, known and documented UGT1A1 deficiency, and previous or concurrent cancer with a distinct primary site, other than gastro-oesophageal cancer, within 5 years before randomisation.

### Study procedures

Eligible patients were randomised (Ennov Clinical® software, Ennov, Paris, France) based on prior use of PD-1/PD-L1 and location of tumour (gastro-oesophageal junction versus gastric adenocarcinomas). Participants allocated to arm A (REGIRI) received REGO (160 mg, oral) daily from day (D) 2-8 and D16-22 plus IRI [180 mg/m^2^, intravenous (i.v.) infusion] every 2 weeks (Supplementary Figure S1, available at https://doi.org/10.1016/j.esmoop.2025.105096).^29^ Patients in arm B (IRI) received IRI (180 mg/m^2^, i.v. infusion) every 2 weeks. Patients were eligible for repeated 4-week treatment cycles (C) in the absence of disease progression, undue adverse events (AEs), investigator decision, or withdrawal of consent. During study treatment, all patients had visits to assess if treatment could be continued every week (REGIRI arm) or every 2 weeks (IRI arm) for the first 2 months and then every 2 weeks until disease progression. Dose reductions and treatment delays were implemented in the event of toxicity. For patients randomised in the REGIRI arm, if REGO or IRI was permanently discontinued, the other study treatment was continued if patient’s condition allowed it.

### Outcomes

Primary endpoint was OS (time from randomisation until death from any cause). Secondary endpoints included PFS (time from randomisation until disease progression or death from any cause, whichever occurred first), ORR [percentage of complete response (CR) or partial response (PR) as best overall response], disease control rate [DCR: percentage of CR, PR, or stable disease (SD)], quality of life (QoL), and safety. For ORR and DCR, patients who discontinued treatment without a tumour assessment were considered non-responders. Efficacy endpoints were evaluated locally according to RECIST v1.1 by computed tomography scan at baseline and then every 8 weeks from treatment initiation until disease progression. All patients had an end-of-treatment visit, and long-term follow-up visits every 3 months for the first 2 years and then every 6 months for 3 years. Safety evaluation was based on the occurrence of AEs graded according to the Common Terminology Criteria for Adverse Events v5.0. Patient-reported outcomes were assessed at baseline, every 8 weeks, and at the end of treatment using the European Organisation for Research and Treatment of Cancer Core QoL Questionnaire (QLQ-C30), monitoring functioning, symptoms, and global health status, with the QLQ-OG25 questionnaire assessing supplementary symptoms. Changes in score were considered to be clinically relevant if there was a >10-point difference from baseline. A *post hoc* analysis was also undertaken to assess ECOG PS time to deterioration defined as the time from randomisation to the date of ECOG PS ≥2 or death from any cause.

### Ancillary studies

For the pharmacogenetic study, blood samples were collected from patients in both arms at C1D1 before IRI infusion. rs9344 (c.723G>A, p.Pro241=) single nucleotide polymorphism (SNP) in the *CCND1* was analysed.^30^^,^^31^ For the pharmacokinetic (PK) study, blood samples from patients allocated to the REGIRI arm were collected at C1D1 (before and at the end of IRI infusion), C1D2 (24 h after the end of IRI infusion and before REGO administration), and then post-dose at C1D8, C1D15, C2D1, C2D8, and C2D15. PK parameters of IRI, REGO, and their active metabolites (SN-38, and M-2 and M-5, respectively) were quantified by a validated liquid chromatography–tandem mass spectrometry method. The prognostic value of PK parameters of REGO was assessed for survival: OS_REGO_ and PFS_REGO_ were, respectively, defined as the time from C1D8 to death due to any cause or disease progression (radiological or clinical) or death, whichever occurred first.

### UGT1A1 genotyping

DNAs were amplified by PCR with the AllTaq Master Mix Kit (Qiagen, Hilden, Germany), using specific fluorescent primers targeting the TATA box-containing region of the *UGT1A1* gene. *UGT1A1* genotyping was obtained by analysing fluorescent amplification products by capillary electrophoresis on the SeqStudio™ Genetic Analyzer System (Thermo Fisher Scientific, Waltham, MA). Results were analysed using GeneMapper V6 software (Thermo Fisher Scientific, Waltham, MA).

### Statistical analysis

To detect a gain of 4 months in median OS corresponding to a hazard ratio (HR) of 0.60, considering the inclusion of 36 patients/year, 122 events were necessary for a power of 80% at a two-sided α risk of 5%. With 10% additional patients (to account for loss of follow-up), 154 patients were required. Sample size calculation was carried out using East 5.

A safety interim analysis was planned after the first 38 patients in the REGIRI arm had received at least one cycle of treatment or discontinued their treatment due to toxicity. An incidence of diarrhoea [grade (G) 3-4] of ≥40% was considered unacceptable toxicity. An efficacy interim analysis was planned after 40 OS events that allowed to reject the null hypothesis if *P* value ≤0.0002 or the alternative hypothesis if *P* value ≥0.969 according to the O’Brien–Fleming boundaries. The study was stopped prematurely and the database locked on 23 May 2022, at which time 79 deaths had occurred.

Efficacy analyses were based on the intention-to-treat (ITT) principle, and safety analyses were done separately on treated patients. OS and PFS were estimated by the Kaplan–Meier method and compared using a two-sided stratified log-rank test. The analysis was stratified by prior use of PD-1/PD-L1 and location of tumour. A Cox proportional hazards model was used to evaluate the effect size of treatment and the effects of prognostic factors in univariate analyses, expressed by HR and its 95% confidence interval (CI). The proportional hazards assumption was verified by the Schoenfeld residual method. ORR and DCR were compared between arms using the χ^2^ or Fisher’s exact test. Health-related QoL scores were described for each assessment and compared between arms using the rank sum test. The association between PK parameters and outcomes were estimated using the Cox proportional hazards regression model with HR and their 95% CI, and compared using the likelihood ratio test. Categorical variables were compared using the χ^2^ or Fisher’s exact test, and continuous variables using the Wilcoxon test. All statistical tests were two-sided and the significance threshold was set at 5%. All analyses were done using SAS v9.4 software (SAS Institute, Cary, NC).

## Results

### Patient characteristics

Between February 2019 and September 2021, 89 patients were included by 22 French participating centres; 44 patients were randomised to the experimental arm (arm A; REGIRI) and 45 to the control arm (arm B; IRI) (Supplementary Figure S2, available at https://doi.org/10.1016/j.esmoop.2025.105096). All patients were considered for the efficacy analysis while two patients in the IRI arm were not treated (one withdrawal; one wrong inclusion) and therefore excluded from the safety analysis. After review of the planned interim efficacy analysis results, the study was stopped prematurely due to the limited efficacy in terms of OS (primary endpoint) and significant toxicities reported in the experimental arm. The majority of patients (86.5%) were men and median age was 62 years (range 28-82 years). Characteristics at inclusion were well balanced between arms except for taxane first-line treatment (Table 1). All patients had previously received fluoropyrimidine and platinum-based chemotherapy, 38.2% patients were treated with taxanes (47.7% in the REGIRI arm and 28.9% in the IRI arm), and 4.5% with an anti-PD-1 (4.6% and 4.4%). The majority of primary tumours (67.4%) were localised in the gastro-oesophageal junction; 60.7% patients had synchronous metastases, and 22.8% were HER2-positive. In total, 29.2% patients had prior gastrectomy.

**Table 1:** Patient characteristics

	Arm A: REGIRI	Arm B: IRI	All
	*n* = 44	*n* = 45	*n* = 89
Age, years			
*n*/missing	44/0	45/0	89/0
Median (min-max)	62 (34-82)	60 (28-80)	62 (28-82)
≤65, *n* (%)	26 (59.09)	27 (60.00)	53 (59.55)
>65, *n* (%)	18 (40.91)	18 (40.00)	36 (40.45)
Sex, *n* (%)			
Male	40 (90.91)	37 (82.22)	77 (86.52)
Female	4 (9.09)	8 (17.78)	12 (13.48)
ECOG PS, *n* (%)			
0	19 (43.2)	17 (37.8)	36 (40.4)
1	25 (56.8)	28 (62.2)	53 (59.6)
Primary tumour assessment, *n* (%)			
Location of tumour			
Gastro-oesophageal junction adenocarcinoma	30 (68.18)	30 (66.67)	60 (67.42)
Gastric adenocarcinoma	14 (31.82)	15 (33.33)	29 (32.58)
Siewert–Stein classification			
Type II: adenocarcinoma of the real cardia	24 (80.00)	22 (73.33)	46 (76.67)
Type III: adenocarcinoma of the subcardial stomach	6 (20.00)	8 (26.67)	14 (23.33)
Histological type, *n* (%)			
Lieberkuhnien adenocarcinoma	22 (50.00)	27 (60.00)	49 (55.06)
Mucinous adenocarcinoma	1 (2.27)	1 (2.22)	2 (2.25)
Independent cells adenocarcinoma	7 (15.91)	7 (15.56)	14 (15.73)
Other	14 (31.82)	10 (22.22)	24 (26.97)
MSI status, *n* (%)			
Missing	26	21	47
MSI	0 (0.00)	2 (8.33)	2 (4.76)
MSS	18 (100.00)	22 (91.67)	40 (95.24)
HER2 status, *n* (%)			
Missing	6	4	10
HER2+	9 (23.68)	9 (21.95)	18 (22.78)
HER2−	29 (76.32)	32 (78.05)	61 (77.22)
Metastatic tumour assessment, *n* (%)			
Synchronous metastases	28 (63.64)	26 (57.78)	54 (60.67)
Metastases location^a^			
Peritoneum	14 (31.82)	16 (35.56)	30 (33.71)
Liver	21 (47.73)	23 (51.11)	44 (49.44)
Lung	8 (18.18)	12 (26.67)	20 (22.47)
Stomach	3 (6.82)	2 (4.44)	5 (5.62)
Bone	7 (15.91)	8 (17.78)	15 (16.85)
Distant lymph node	23 (52.27)	18 (40.00)	41 (46.07)
Kidney	0 (0.00)	1 (2.22)	1 (1.12)
Brain	0 (0.00)	1 (2.22)	1 (1.12)
Other	7 (15.91)	6 (13.33)	13 (14.61)
Prior cancer therapies, *n* (%)			
Chemotherapy^b^	44 (100.00)	45 (100.00)	89 (100.00)
Fluoropyrimidine and platinum-based chemotherapy	44 (100.00)	45 (100.00)	89 (100.00)
Taxanes	21 (47.73)	13 (28.89)	34 (38.20)
Radiotherapy, *n* (%)	9 (20.45)	6 (13.33)	15 (16.85)
Surgery:	17 (38.64)	18 (40.00)	35 (39.33)
Gastrectomy	12 (27.27)	14 (31.11)	26 (29.21)
Other cancer therapy, *n* (%)	6 (13.64)	9 (20.00)	15 (16.85)
Anti-PD-1	2 (4.55)	2 (4.44)	4 (4.49)
Trastuzumab	4 (9.09)	5 (11.11)	9 (10.11)
Zolbetuximab	0 (0.00)	1 (2.22)	1 (1.12)
CHIP mitomycin	0 (0.00)	1 (2.22)	1 (1.12)

ECOG PS, Eastern Cooperative Oncology Group performance status; HER2, human epidermal growth factor receptor 2; MSI, microsatellite instability; MSS, microsatellite stability; PD-1, programmed cell death protein 1.

a Several locations possible.

b Several chemotherapies possible.

### Efficacy

**Figure 1:**
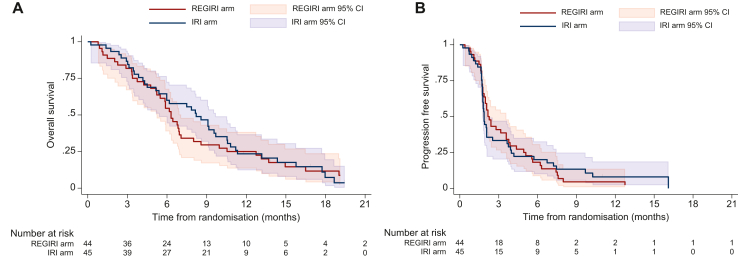
**OS (A) and PFS (B) according to the treatment arms.** CI, confidence interval; HR, hazard ratio; IRI, irinotecan; OS, overall survival; PD-1, programmed cell death protein 1; PD-L1, programmed death-ligand 1; PFS, progression-free survival. ^a^Stratified log-rank test on prior use of PD-1/PD-L1 inhibitors and the location of tumour.

Median follow-up was 19.4 months (95% CI 16.8-29.9 months) and 79 patients had died: 39 (88.6%) in the REGIRI arm versus 40 (88.9%) in the IRI arm, 70 following disease progression (*n* = 32; 82.1% versus *n* = 38; 95.0%), 8 due to toxicity (*n* = 6; 15.4% versus *n* = 2; 5.0%), and 1 in the REGIRI arm due to an unknown reason. Median OS was 6.3 months (95% CI 5.2-7.1 months) versus 8.2 months (95% CI 5.4-9.7 months) (HR 1.11, 95% CI 0.70-1.74, *P* = 0.66), and 6-month survival rate 54.6% (95% CI 38.8% to 67.8%) versus 60.0% (95% CI 44.3% to 72.6%) (Figure 1). PFS events were reported for 85 patients and median PFS was 2.2 months (95% CI 1.8-3.6 months) versus 1.9 months (95% CI 1.7-2.1 months). Among the ITT population, seven patients were not assessable for response (no tumour evaluation; Table 2). Of 82 assessable patients, 7 had PR and 13 SD in the REGIRI arm, versus 1 CR, 5 PR, and 9 SD in the IRI arm. ORR and DCR were, respectively, 15.9% versus 13.3% and 45.5% versus 33.3% (*P* = 0.26). In the univariate analysis, patients who underwent gastrectomy had a significantly better survival outcome with a 6-month survival rate of 76.9% compared with 49.2% for unresected patients (*P* = 0.0133). No prognostic factors were found for PFS (data not shown). Subgroup analyses show that the presence of hepatic metastases was associated with numerically lower ORR in both arms; this trend was not observed in terms of median PFS (Supplementary Table S1, available at https://doi.org/10.1016/j.esmoop.2025.105096).

**Table 2:** Summary of ORR and DCR

	Arm A: REGIRI	Arm B: IRI	All
	*n* = 44	*n* = 45	*n* = 89
Best overall response, *n* (%)			
Not evaluable^a^	3 (6.82)	4 (8.89)	7 (7.87)
Complete response (CR)	0 (0.00)	1 (2.22)	1 (1.12)
Partial response (PR)	7 (15.91)	5 (11.11)	12 (13.48)
Stable disease (SD)	13 (29.55)	9 (20.00)	22 (24.72)
Progressive disease (PD)	21 (47.73)	26 (57.78)	47 (52.81)
Objective response rate (ORR), *n* (%)			
CR + PR	7 (15.91)	6 (13.33)	13 (14.61)
Disease control rate (DCR), *n* (%)			
CR + PR + SD	20 (45.45)	15 (33.33)	35 (39.33)

a No tumour assessment.

### Compliance

Median number of cycles was 3 (range 1-18) and 2 (range 1-12) in the REGIRI and IRI arms, respectively (Supplementary Table S2, available at https://doi.org/10.1016/j.esmoop.2025.105096). In the IRI arm, median relative dose intensity (RDI) was 0.97 (range 0.61-1.06), 86.1% of patients received at least 80% of the theoretical dose of IRI, 27.9% had at least one IRI administration delayed, 33.3% of which for toxicity, and 27.9% had at least one IRI dose adjustment, 66.7% of which for toxicity. In the REGIRI arm, median RDI was 0.96 (range 0.41-1.11) and 0.97 (0.39-1.08) for IRI and REGO, respectively. 77.3% of patients received at least 80% of the theoretical dose of IRI, 40.9% had at least one IRI administration delayed, 50.0% of which for toxicity, and 40.9% (*n* = 18) had at least one IRI dose adjustment, 83.3% (*n* = 15) of which for toxicity. In this arm, 77.3% (*n* = 34) of patients received at least 80% of the theoretical dose of REGO, 45.5% (*n* = 20) had at least one REGO administration delayed, 50.0% (*n* = 10) of which for toxicity, and 59.1% (*n* = 26) had at least one REGO dose adjustment, 61.5% (*n* = 16) of which for toxicity.

### Safety

In the safety population, 52.3% patients in the REGIRI arm versus 23.3% patients in the IRI arm had at least one G ≥3 treatment-related AE (Supplementary Table S3, available at https://doi.org/10.1016/j.esmoop.2025.105096). Most frequent G ≥3 treatment-related AEs were diarrhoea (18.2% in the REGIRI arm versus 7.0% in the IRI arm), hypertension (9.1% versus 0.0%), asthenia (6.8% versus 0.0%), febrile neutropenia (6.8% versus 0.0%), neutropenia (6.8% versus 11.6%), and weight decreased (6.8% versus 0.0%) (Table 3). Five AEs resulting in deaths were considered related to the treatments, four in the REGIRI arm [thrombotic microangiopathy (C1D15), sepsis complicated with respiratory decompensation (C1D15), diarrhoea (C3D1), and pulmonary embolism (C4D15)] and one in the IRI arm [primary tumour perforation (C1D1)].

**Table 3:** Treatment-related G ≥3 AEs classified by SOC^a^

SOC name prefer term name	Arm A: REGIRI		Arm B: IRI		All	
	Patients *n* = 44 *n* (%)	AE *n* = 80	Patients *n* = 43 *n* (%)	AE *n* = 22	Patients *n* = 87 *n* (%)	AE *n* = 102
Blood and lymphatic system disorders	9 (20.5)	12	6 (14.0)	10	15 (17.2)	22
Anaemia	0 (0.0)	0	1 (2.3)	1	1 (1.1)	1
Febrile neutropenia	3 (6.8)	4	0 (0.0)	0	3 (3.4)	4
Leukopenia	1 (2.3)	1	0 (0.0)	0	1 (1.1)	1
Lymphopenia	2 (4.5)	3	1 (2.3)	2	3 (3.4)	5
Neutropenia	3 (6.8)	3	5 (11.6)	7	8 (9.2)	10
Thrombotic microangiopathy	1 (2.3)	1	0 (0.0)	0	1 (1.1)	1
Gastrointestinal disorders	11 (25.0)	26	5 (11.6)	6	16 (18.4)	32
Abdominal pain	2 (4.5)	3	0 (0.0)	0	2 (2.3)	3
Diarrhoea	8 (18.2)	16	3 (7.0)	3	11 (12.6)	19
Nausea	2 (4.5)	5	1 (2.3)	2	3 (3.4)	7
Retroperitoneal haematoma	1 (2.3)	1	0 (0.0)	0	1 (1.1)	1
Vomiting	1 (2.3)	1	1 (2.3)	1	2 (2.3)	2
General disorders and administration site conditions	6 (13.6)	14	0 (0.0)	0	6 (6.9)	14
Asthenia	3 (6.8)	6	0 (0.0)	0	3 (3.4)	6
Fatigue	2 (4.5)	7	0 (0.0)	0	2 (2.3)	7
General physical health deterioration	1 (2.3)	1	0 (0.0)	0	1 (1.1)	1
Hepatobiliary disorders	1 (2.3)	5	0 (0.0)	0	1 (1.1)	5
Cholestasis	1 (2.3)	1	0 (0.0)	0	1 (1.1)	1
Hepatic cytolysis	1 (2.3)	4	0 (0.0)	0	1 (1.1)	4
Immune system disorders	1 (2.3)	1	0 (0.0)	0	1 (1.1)	1
Anaphylactic shock	1 (2.3)	1	0 (0.0)	0	1 (1.1)	1
Infections and infestations	1 (2.3)	1	0 (0.0)	0	1 (1.1)	1
Sepsis	1 (2.3)	1	0 (0.0)	0	1 (1.1)	1
Investigations	3 (6.8)	3	2 (4.7)	3	5 (5.7)	6
Blood alkaline phosphatase increased	0 (0.0)	0	1 (2.3)	1	1 (1.1)	1
γ-Glutamyltransferase increased	0 (0.0)	0	2 (4.7)	2	2 (2.3)	2
Weight decreased	3 (6.8)	3	0 (0.0)	0	3 (3.4)	3
Metabolism and nutrition disorders	3 (6.8)	5	0 (0.0)	0	3 (3.4)	5
Decreased appetite	2 (4.5)	4	0 (0.0)	0	2 (2.3)	4
Dehydration	1 (2.3)	1	0 (0.0)	0	1 (1.1)	1
Neoplasms benign, malignant and unspecified (including cysts and polyps)	0 (0.0)	0	1 (2.3)	1	1 (1.1)	1
Tumour perforation	0 (0.0)	0	1 (2.3)	1	1 (1.1)	1
Nervous system disorders	0 (0.0)	0	1 (2.3)	1	1 (1.1)	1
Neuropathy peripheral	0 (0.0)	0	1 (2.3)	1	1 (1.1)	1
Renal and urinary disorders	1 (2.3)	1	1 (2.3)	1	2 (2.3)	2
Acute kidney injury	1 (2.3)	1	0 (0.0)	0	1 (1.1)	1
Renal failure	0 (0.0)	0	1 (2.3)	1	1 (1.1)	1
Skin and subcutaneous tissue disorders	2 (4.5)	2	0 (0.0)	0	2 (2.3)	2
Alopecia	1 (2.3)	1	0 (0.0)	0	1 (1.1)	1
Palmar-plantar erythrodysesthesia syndrome	1 (2.3)	1	0 (0.0)	0	1 (1.1)	1
Vascular disorders	5 (11.4)	10	0 (0.0)	0	5 (5.7)	10
Hypertension	4 (9.1)	7	0 (0.0)	0	4 (4.6)	7
Pulmonary embolism	2 (4.5)	3	0 (0.0)	0	2 (2.3)	3

AE, adverse event.

a The most common treatment-related G ≥3 AEs are highlighted in grey.

### Time to deterioration in ECOG PS and QoL

**Figure 2:**
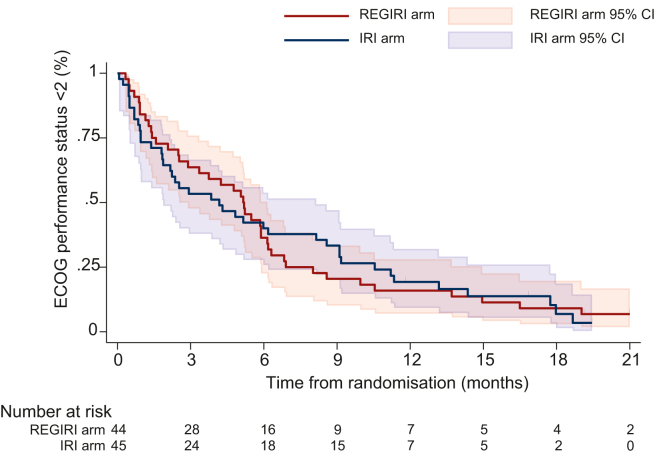
**Time to deterioration in ECOG PS score to ≥2.** Time to deterioration of ECOG PS was defined as a decline in ECOG PS ≥2. The *y*-axis shows the percentage of patients whose ECOG PS <2. Median time to deterioration (months) is shown, along with the total number of patients at risk. CI, confidence interval; ECOG PS, Eastern Cooperative Oncology Group performance status; IRI, irinotecan.

Median time to ECOG PS deterioration ≥2 was 5.2 months (range 2.5-6.1 months) in the REGIRI arm and 4.2 months (range 1.9-8.1 months) in the IRI arm (Figure 2). QLQ-C30/OG25 questionnaire completion rates ranged from 80% to 100% through the five first cycles of treatment (Supplementary Figure S3, available at https://doi.org/10.1016/j.esmoop.2025.105096). Given a good health status translates to values ≥70, scores revealed poor health condition at baseline with mean global health status scores of 62.6 in the REGIRI arm versus 57.2 in the IRI arm (Supplementary Table S4, available at https://doi.org/10.1016/j.esmoop.2025.105096). Using differences in scale of ≥10 points to indicate clinical relevance, evolution between baseline and cycle 3 showed an increase in constipation in the IRI arm but not in the REGIRI arm (Supplementary Table S5, available at https://doi.org/10.1016/j.esmoop.2025.105096). There was no difference between treatment groups on the other subscales (Supplementary Figures S4 and S5, available at https://doi.org/10.1016/j.esmoop.2025.105096).

### *CCND1* polymorphism

Among the 72 patients analysed for *CCND1* SNP, 26.4% were homozygous for the A allele of the gene (36.1% in the REGIRI arm versus 16.7% in the IRI arm), 20.8% patients were G/G (16.7% versus 25.0%), and 52.8% patients were heterozygous (47.2% versus 58.3%). No prognostic effect of cyclin D1 genotype was detected on OS and PFS (Supplementary Table S6, available at https://doi.org/10.1016/j.esmoop.2025.105096).

### PK ancillary study

PK data were available for 35 patients in the REGIRI arm who had at least one blood sample collected (Supplementary Table S7, available at https://doi.org/10.1016/j.esmoop.2025.105096). An accumulation of SN-38 was observed in that arm at C2D15 compared with C1D15 with 26.1 (±14.9) versus 19.5 (±2.3) ng/ml (*P* = 0.03), while SN-38 concentrations at C1D1 and C2D1 after IRI intake remained similar. No difference was observed between concentrations of REGO and its metabolites measured at C1D8 and C2D8. According to baseline characteristics, IRI concentrations were statistically higher in overweight/obese patients at C1D15 and in HER2-negative patients at C2D15, while SN-38 concentrations were more elevated at C1D1 in patients who did not undergo gastrectomy.

Out of the 35 patients, 28 had at least one G ≥3 AE (Supplementary Table S8, available at https://doi.org/10.1016/j.esmoop.2025.105096). In these patients, concentrations of IRI but not REGO or their metabolites were higher at C2D1 than in patients without G ≥3 AEs. In this population, six patients had G5 AEs. These patients had higher concentrations of IRI at C1D1 and elevated SN-38/IRI ratio at C1D15, while the concentrations of REGO and its metabolites were similar.

The prognostic value of REGO PK parameters was studied in the 28 patients randomised in the REGIRI arm who had at least one blood sample at C1D8. Median follow-up for this population was 26.6 months (95% CI 13.8-29.9 months), median OS_REGO_ 6.7 months (95% CI 5.5-10.5 months), and median PFS_REGO_ 2.3 months (95% CI 1.7-3.6 months) (Supplementary Table S6, available at https://doi.org/10.1016/j.esmoop.2025.105096). No PK parameters were prognostic for OS_REGO_ and PFS_REGO_ (Supplementary Table S9, available at https://doi.org/10.1016/j.esmoop.2025.105096). BMI and previous gastrectomy, however, are prognostic of PFS_REGO_ in this population.

### *UGT1A1* polymorphism

Genotyping of the UGT1A1 promoter sequence was carried out on 72 samples: 43.1% (*n* = 31) patients were homozygous *UGT1A1∗1* (38.9% in the REGIRI arm and 47.2% in the IRI arm), 13.9% homozygous *UGT1A1∗28* (11.1% and 16.7%), and 43.1% heterozygous *UGT1A1∗1∗28* (50.0% and 36.1%). The allelic frequencies were consistent with those reported in the literature.^32^, ^33^, ^34^ Among the four G5 AEs reported in the REGIRI arm, two were homozygous *UGT1A1∗28* (REGO-related thrombotic microangiopathy; IRI-related sepsis), and two were heterozygous *UGT1A1∗1∗28* (IRI-related pulmonary embolism; IRI/REGO-related diarrhoea), while the one G5 AE reported in the IRI arm was homozygous *UGT1A1∗1* (tumour perforation). We noted that the events occurred in the REGIRI arm at C1D15 for the *UGT1A1∗28* patients and at later cycles for the heterozygous patients (C3D1 and C4D15). In this arm, mean number of cycles was 2.5 (standard deviation 1.29), 3.56 (2.12), and 5.14 (4.99) in the *UGT1A1∗28*, heterozygous, and *UGT1A1∗1* patients, respectively (Supplementary Table S10, available at https://doi.org/10.1016/j.esmoop.2025.105096). Also, the last treatment cycle of the G5 septic patient was complicated with febrile neutropenia, a common AE reported in patients receiving IRI (Table 4).

**Table 4:** Description of the AEs reported by patients who received IRI-based therapy in the REGIRI safety population

SOC name prefer term name	*UGT1A1∗1*	*UGT1A1∗28*	*UGT1A1∗1∗28*	*UGT1A1∗1*	*UGT1A1∗28*	*UGT1A1∗1∗28*	All
	*n* = 14 *n* (%)	*n* = 4 *n* (%)	*n* = 18 *n* (%)	*n* = 17 *n* (%)	*n* = 6 *n* (%)	*n* = 13 *n* (%)	*n* = 72 *n* (%)
Blood and lymphatic system disorders	7 (50.00)	2 (50.00)	10 (55.56)	3 (17.65)	5 (83.33)	8 (61.54)	35 (48.61)
Anaemia^a^	4 (28.57)	1 (25.00)	5 (27.78)	3 (17.65)	2 (33.33)	6 (46.15)	21 (29.17)
Febrile neutropenia^b^	3 (21.43)	0 (0.00)	1 (5.56)	0 (0.00)	0 (0.00)	0 (0.00)	4 (5.56)
Leukopenia^a^	0 (0.00)	0 (0.00)	2 (11.11)	0 (0.00)	0 (0.00)	0 (0.00)	2 (2.78)
Neutropenia^a^	2 (14.29)	1 (25.00)	8 (44.44)	1 (5.88)	4 (66.67)	2 (15.38)	18 (25.00)
Gastrointestinal disorders	14 (100.00)	4 (100.00)	15 (83.33)	15 (88.24)	4 (66.67)	9 (69.23)	61 (84.72)
Diarrhoea^a^	11 (78.57)	3 (75.00)	15 (83.33)	14 (82.35)	2 (33.33)	7 (53.85)	52 (72.22)
Nausea^a^	8 (57.14)	2 (50.00)	8 (44.44)	10 (58.82)	4 (66.67)	6 (46.15)	38 (52.78)
Vomiting^a^	3 (21.43)	2 (50.00)	6 (33.33)	5 (29.41)	1 (16.67)	4 (30.77)	21 (29.17)
General disorders and administration site conditions	11 (78.57)	3 (75.00)	13 (72.22)	8 (47.06)	3 (50.00)	7 (53.85)	45 (62.50)
Asthenia^b^	10 (71.43)	1 (25.00)	10 (55.56)	6 (35.29)	2 (33.33)	6 (46.15)	35 (48.61)
Infections and infestations	0 (0.00)	1 (25.00)	4 (22.22)	1 (5.88)	1 (16.67)	0 (0.00)	7 (9.72)
Sepsis^b^	0 (0.00)	1 (25.00)	0 (0.00)	0 (0.00)	0 (0.00)	0 (0.00)	1 (1.39)
Metabolism and nutrition disorders	6 (42.86)	3 (75.00)	6 (33.33)	4 (23.53)	2 (33.33)	5 (38.46)	26 (36.11)
Decreased appetite^b^	4 (28.57)	2 (50.00)	4 (22.22)	4 (23.53)	1 (16.67)	3 (23.08)	18 (25.00)
Skin and subcutaneous tissue disorders	8 (57.14)	1 (25.00)	7 (38.89)	1 (5.88)	2 (33.33)	2 (15.38)	21 (29.17)
Alopecia^a^	5 (35.71)	1 (25.00)	6 (33.33)	1 (5.88)	1 (16.67)	1 (7.69)	15 (20.83)
Vascular disorders	3 (21.43)	1 (25.00)	3 (16.67)	0 (0.00)	1 (16.67)	0 (0.00)	8 (11.11)
Pulmonary embolism^a^	1 (7.14)	0 (0.00)	1 (5.56)	0 (0.00)	0 (0.00)	0 (0.00)	2 (2.78)

IRI, irinotecan; SOC, .

a Very common (IRI-related AE reported in ≥10% of the patients enrolled in clinical studies).

b Common (IRI-related AE reported in ≥1% to <10% of the patients enrolled in clinical studies).

## Discussion

REGO is an oral multikinase inhibitor indicated in the treatment of various solid tumours including metastatic colorectal cancer (mCRC), locally advanced, unresectable or metastatic gastrointestinal stromal tumours, and hepatocellular carcinoma (HCC) previously treated with sorafenib.^35^, ^36^, ^37^, ^38^ The phase II INTEGRATE trial first showed promising efficacy of REGO versus placebo in the treatment of patients with advanced GCs pretreated with two or fewer lines of chemotherapy. Indeed, it demonstrated a significant improvement in PFS (2.6 versus 0.9 months, HR 0.40, 95% CI 0.28-0.59, *P* = 0.01) and a non-significant numerical improvement in OS (5.8 versus 4.5 months).^27^ Meanwhile, the double-blind, placebo-controlled, phase III INTEGRATE IIa trial comparing REGO with placebo using a 2 : 1 randomisation, stratified by tumour location (gastro-oesophageal junction versus gastric), geographic region (Asia versus rest of the world), and prior VEGF inhibitors, examined REGO efficacy in terms of OS. Median OS for REGO versus placebo was 4.5 versus 4.0 months (HR 0.70, 95% CI 0.53-0.92, *P* = 0.011) in the study population and median PFS, 1.8 versus 1.6 months (HR 0.52, 95% CI 0.40-0.69, *P* < 0.0001).^28^ Before anti-PD-1 approval in France, the comparative PRODIGE58-UCGI35-REGIRI study was designed to detect the superiority of REGO plus IRI versus IRI alone on OS as 2L treatment in mGA patients, using a 1 : 1 randomisation design and stratification by prior use of PD-1/PD-L1 inhibitors and location of tumour. After review of the planned interim efficacy analysis results, the study was stopped prematurely in 2021 due to the limited efficacy in terms of OS (primary endpoint) and the significant toxicities reported in the experimental arm. Therefore, all our findings should be considered exploratory. At the time of the final analysis, with a median follow-up of 19.4 months (95% CI 16.8-29.9 months), median OS was 6.3 months (95% CI 5.2-7.1 months) in the REGIRI arm and 8.2 months (95% CI 5.4-9.7 months) in the IRI arm. Of note, patients in the IRI arm achieved longer median OS than those included in initial phase III studies which explored the efficacy of IRI monotherapy administered weekly (2.6 months, 95% CI 2.4-2.8 months), every 2 weeks (6.5 months, 95% CI 4.5-8.5 months), and every 3 weeks (4.0 months, 95% CI 3.6-7.5 months).^20^^,^^39^^,^^40^ Our results are in line with those reported in the WJOG 4007 trial, however, in which median OS was, respectively, 8.4 months and 9.5 months (HR 1.13, 95% CI 0.86-1.49, *P* = 0.38) with 2L IRI and paclitaxel in Japanese patients.^18^ The median OS observed in the REGIRI arm was also comparable with that of 2L or 3L REGO in the INTEGRATE study (5.8 months, 95% CI 4.4-6.8 months). Concerning secondary endpoints, median PFS was consistent with data from the previously mentioned trials.^20^^,^^39^^,^^40^ We showed an ORR of 15.9% versus 13.3%, and a DCR of 45.5% versus 33.3% in the REGIRI versus IRI arm. In the Korean trials, OR ranged from 8% to 20%, and overall control rate was estimated at 42.9%.^39^^,^^40^ In the German AIO study,^20^ IRI did not provide OR but an improvement in tumour-related symptoms. Regarding the preservation of patients’ functional status and QoL, which is a key goal in the treatment of GCs, often allowing them to receive further treatment, the results presented here reported no QoL degradation in the REGIRI arm. Furthermore, QLQ-C30/QLQ-OG25 data supported the time to deterioration in ECOG PS analysis with a higher degree of impairment after cycle 5 in both arms. Interestingly, patients treated with REGIRI did not experience an acceleration in time to deterioration over those treated with IRI alone. Finally, as despite the development of better treatment options, the prognosis of patients with advanced GCs remains poor, we also investigated the values of prognostic factors. Our exploratory analysis identified, for the first time, gastrectomy as a potential prognostic factor for OS while *CCND1* SNP had no impact even though rs9344 (c.723G>A, p.Pro241=) was associated with favourable outcomes in mCRC treated with IRI/sorafenib or IRI/cetuximab combinations.^30^^,^^31^^,^^41^^,^^42^ Nevertheless, the treatment effect of REGO plus IRI was similar for OS, irrespective of gastrectomy.

Regarding AEs, previously published studies demonstrated an acceptable safety profile with REGO monotherapy or REGO plus fluoropyrimidine and platinum or IRI-based compound in refractory mCRC, HCC, and GC.^28^^,^^29^^,^^35^^,^^37^^,^^43^ Here, the toxicity was significant and biased against the experimental arm with five G5 AEs considered related to the treatment, four versus one in the REGIRI versus IRI arm, and 52.3% versus 23.3% patients who had at least one G ≥3 treatment-related AE. Of note, the use of taxanes in the frontline setting was unbalanced between arms (48% versus 29%). Common toxicities associated with taxane treatment in gastrointestinal cancers include gastrointestinal complications such as diarrhoea and mucositis. In this trial, however, the toxic deaths were not necessarily related to gastrointestinal toxicities since only one was due to diarrhoea. The previous RAINBOW and REGARD trials did not report an increase in pulmonary embolism, which in the PRODIGE58-UCGI35-REGIRI study may have been more related to the anti-angiogenic component.^22^, ^23^, ^24^, ^25^ The perforation observed in the IRI arm is also more of a complication expected from anti-angiogenic therapy. The most common G ≥3 AEs reported were diarrhoea and asthenia, frequently encountered with both REGO and IRI, and weight loss, neutropenia, and febrile neutropenia commonly associated with IRI.^44^, ^45^, ^46^ These AEs were usually observed when using these drugs. Moreover, the combination of REGO given sequentially as a 1 week out of 2 schedule to FOLFOX or FOLFIRI has shown acceptable tolerability in mCRC, which is why we chose this schedule in combination with IRI.^29^ Despite this precaution, drug interaction was still possible since REGO and its metabolites are strong inhibitors of the glucuronidation mediated by UGT1A1 and therefore, could have increased systemic exposure to the enzyme substrates, including SN-38. Such interaction was previously described with sorafenib, an agent structurally similar to REGO.^47^^,^^48^ Furthermore, if patients with documented *UGT1A1* deficiency were excluded from the study, genotyping was not routinely carried out before the administration of IRI. UGT1A1 being the predominant factor of IRI-induced cytotoxicity and AEs,^49^ the reported increase in toxicity may have therefore resulted from drug accumulation. Consequent to the relatively low number of *UGT1A1∗28* patients, conclusions are difficult to draw. Nevertheless, as previously reported,^34^ our data seemed to point out an association between *UGT1A1∗28* polymorphism and neutropenia. Furthermore, out of the four G5 AEs reported in the REGIRI arm, two were homozygous *UGT1A1∗28*. The other two G5 AEs were heterozygous but still likely caused by IRI. Of note, the events were reported early on during treatment for the *UGT1A1∗28* homozygous patients and later on for the heterozygous patients. Incidentally, PK ancillary studies revealed an accumulation of SN-38 between C1 and C2 and higher concentrations of IRI at C2D1 in patients who reported at least one G ≥3 AE. Similarly, elevated concentrations of IRI and SN-38 seemed detrimental in patients who did not undergo gastrectomy or were overweight/obese, as these baseline characteristics were found associated with worse PFS. Meanwhile, no differences were detected for concentrations of REGO and its metabolites. These concentrations were in line with those observed in the TEXCAN phase II trial, but contrary to the TEXCAN study, no accumulation of M-2 was observed and REGO PK parameters were not found prognostic of survival.^50^ If the limited number of patients complicated the assessment of the joint IRI/REGO PK profiles, our results are a clear indication that the pretherapeutic systematic analysis of commonly encountered *UGT1A1* variants and a progressive increase of IRI depending on patients *UGT1A1* genotype could help manage the risk of IRI-induced severe toxicities.

### Conclusion

The PRODIGE58-UCGI35-REGIRI study failed to show an improvement in terms of OS with REGO plus IRI and was discontinued prematurely due to an unfavourable benefit/risk ratio. The exploratory analyses presented in this article serve as a basis for optimising precautionary use of REGO and supporting further research. Still, several recent clinical trials have demonstrated that REGO is active in advanced GCs and is currently evaluated in 3L in combination with nivolumab in the phase III INTEGRATE IIb trial (NCT04879368). With PD-1 antibodies now approved for GCs, REGO use following prior anti-PD-1 exposure might also emerge as a promising strategy in carefully selected GC patients. Among anti-angiogenic drugs, ramucirumab, approved for the treatment of mGC patients, plus IRI also showed anticancer activity and a manageable safety profile in 2L Japanese patients with advanced GC.^51^ Taken together, these results highlight the need to investigate the clinical and molecular characteristics of this heterogeneous disease and identify the populations likely to benefit the most from a new developed regimen.
