# Supplementary material for: Regorafenib combined with irinotecan as second-line treatment in metastatic gastro-oesophageal adenocarcinomas: results of PRODIGE 58–UCGI35–REGIRI Unicancer randomised phase II study
Source: ESMO Open. 2025 May 12;10(5):105096. doi: 10.1016/j.esmoop.2025.105096 (PMC12141891; doi:10.1016/j.esmoop.2025.105096)
Supplement: Supplementary Data [file mmc1.docx]

Supplementary Material

Figure S1: Study design


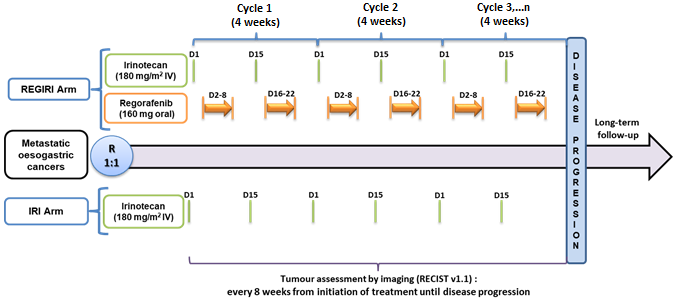


Figure S2: Consort flow chart

**Analysis**

|  |  | Assessed for eligibility  (N=108) |  |  |
| --- | --- | --- | --- | --- |
|  |  |  | N=1 death  N=1 physician decision |  |
| **Enrolment** |  |  | N=17 screen failures | |
|  |  |  |  |  |
|  |  | Randomized  (N=89) |  |  |
|  |  |  |  |  |
|  |  |  |  |  |
| **REGIRI arm (N=44)**  **Allocation**  Received allocated treatment (N=44) | |  | **IRI arm (N=45)**  Received allocated treatment (N=43)  Patient withdrawal (N=1) | |
|  |  |  |  |  |
|  |  |  |  |  |
|  | |  |  | |
|  | |  |  | |
| ITT population (N=44)  Safety population (N=44) | |  | ITT population (N=45)  Safety population (N=43) | |

Table S1: Efficacy subgroup analyses in patients with and without hepatic metastases

|  | **Arm A : REGIRI** | | **Arm B : IRI** | |
| --- | --- | --- | --- | --- |
|  | Hepatic metastases  (n=21) | No hepatic metastases  (n=23) | Hepatic metastases  (n=23) | No hepatic  metastases  (n=22) |
| **ORR (%)** | 10.5% (2/19) | 22.7% (5/22) | 10.5% (2/19) | 18.2% (4/22) |
| Missing | 2 | 1 | 4 | 0 |
| IC95% ORR | [1.3-33.1] | [7.8-45.4] | [1.3-33.1] | [5.2-40.3] |
| **median PFS (months)** | 2.8 | 2.1 | 2.0 | 1.8 |
| IC95% | [1.8-5.1] | [1.7-3.2] | [1.8-2.5] | [1.6-6.7] |
| **median OS (months)** | 6.3 | 6.2 | 7.9 | 8.2 |
| IC95% | [5.191-7.064] | [3.351, 9.955] | [3.450, 9.692] | [5.979, 14.357] |

Table S2: Compliance to study treatments

|  | **ARM A: REGIRI** | | **ARM B: IRI** | |
| --- | --- | --- | --- | --- |
|  | **N=44** | | **N=43** | |
| Nb of treated patients (N [%]) | 44 | (100.00%) | 43 | (95.56%) |
| Nb of administered cycles (median [min; max]) | 3 | (1;18) | 2 | (1;12) |
| Treatment discontinuations (N [%]) | 44 | (100.00%) | 41 | (95.35%) |
| Reason for discontinuation (N [%]) |  |  |  |  |
| Toxicity | 9 | (20.45%) | 5 | (12.20%) |
| Progressive disease | 31 | (70.45%) | 30 | (73.17%) |
| Physician decision | 0 | (0.00%) | 2 | (4.88%) |
| Withdrawal by subject | 2 | (4.55%) | 1 | (2.44%) |
| Death | 2 | (4.55%) | 1 | (2.44%) |
| Other | 0 | (0.00%) | 2 | (4.88%) |
| Treatment reductions (N [%]) |  |  |  |  |
| Regorafenib | 26 | (59.09%) | / | |
| Irinotecan | 18 | (40.91%) | 12 | (27.91%) |
| Dose adjustment due to toxicity |  |  |  |  |
| Regorafenib | 16 | (61.53%) | / | |
| Irinotecan | 15 | (83.33%) | 8 | (66.67%) |
| Treatment delays (N [%]) |  |  |  |  |
| Regorafenib | 20 | (45.45%) | / | |
| Irinotecan | 18 | (40.91%) | 12 | (27.91%) |
| Dose adjustment due to toxicity |  |  |  |  |
| Regorafenib | 10 | (50.00%) | / | |
| Irinotecan | 9 | (50.00%) | 4 | (33.33%) |
| RDI* (median [min; max]) |  |  |  |  |
| Regorafenib | 0.97 | (0.39;1.08) | / | |
| Irinotecan | 0.96 | (0.41;1.11) | 0.97 | (0.61;1.06) |
| RDI*≥80% (median [min; max]) |  |  |  |  |
| Regorafenib | 34 | (77.27%) | / | |
| Irinotecan | 34 | (77.27%) | 37 | (86.1%) |

*RDI: relative dose intensity

Table S3: Summary of AEs

|  | **ARM A: REGIRI** | | **ARM B: IRI** | | **All** | |
| --- | --- | --- | --- | --- | --- | --- |
|  | **N=44** | | **N=43** | | **N=87** | |
| AEs* | 44 | (100.0%) | 43 | (100.0%) | 87 | (100.0%) |
| Treatment-related AEs | 42 | (95.5%) | 38 | (88.4%) | 80 | (92.0%) |
| Grade≥3 AEs | 35 | (79.6%) | 23 | (53.5%) | 58 | (66.7%) |
| Treatment-related AEs | 23 | (52.3%) | 10 | (23.3%) | 33 | (37.9%) |
| SAEs** | 22 | (50.0%) | 17 | (39.5%) | 39 | (44.8%) |
| Treatment-related SAEs |  |  |  |  |  |  |
| Grade 5 AEs | 6 | (13.6%) | 5 | (11.6%) | 11 | (12.6%) |
| Treatment-related AEs | 4 | (9.1%) | 1 | (2.3%) | 5 | (5.7%) |

*AEs adverse events

**SAEs serious adverse events

***NA not applicable

Figure S3: Compliance over time to QLQ-C30 and QLQ-OG25

Compliance at each cycle was based on the number of patients who completed the questionnaires divided by the number of patients still on treatment at each cycle.

Table S4: QLQ-C30 scores at baseline

|  | **Arm A: REGIRI** | | | | | **Arm B: IRI** | | | | | |
| --- | --- | --- | --- | --- | --- | --- | --- | --- | --- | --- | --- |
|  | **N** | **Median** | **Range** | **Mean** | **SD** | **N** | **Median** | **Range** | **Mean** | **SD** |  |
| Global health status | 35 | 66.67 | [8.33;100] | 62.62 | 20.85 | 38 | 54.17 | [25;91.67] | 57.24 | 19.49 |  |

Scores range 0–100. High scores represent better QoL for global QoL/health status.

Figure S4: Evolution of QLQ-C30 scores over time

|  |  |
| --- | --- |
|  |  |
|  |  |

|  |  |
| --- | --- |
|  |  |
|  |  |
|  |  |
|  |  |

Table S5: QLQ-C30 scores evolution between baseline and cycle 3

|  | **ARM A: REGIRI** | | **ARM B: IRI** | | **All** | | **P-value**  **Fisher exact** |
| --- | --- | --- | --- | --- | --- | --- | --- |
|  | **N=20** | | **N=14** | | **N=34** | |  |
| **Constipation** |  |  |  |  |  |  | **P = 0.012** |
| No | 19 | (95.00%) | 8 | (57.14%) | 27 | (79.41%) |  |
| Yes* | 1 | (5.00%) | 6 | (42.86%) | 7 | (20.59%) |  |

*Degradation of QLQ-C30 scores was defined by a >10 points decrease for functional scales and a >10 points increase for symptom scales between baseline and cycle 3.

Figure S5: Evolution of QLQ-OG5 scores over time

|  |  |
| --- | --- |
|  |  |
|  |  |
|  |  |
|  |  |
|  |  |
|  |  |
|  |  |

Table S6: Impact of cyclin D1 polymorphism on OS and PFS

| **Cyclin D1 Genotype** | **Event/Total** | **6-month estimates**  **(95% CI)**^KM^ | **Unstratified HR**  **(95% CI)**^Cox^ | **p-value** |
| --- | --- | --- | --- | --- |
| **OS** |  |  |  | 0.7338 |
| A/A | 17/19 | 63.2 (44.8-89.0%) | 1.10 (0.63-1.93) |  |
| A/G or G/G | 47/53 | 54.7 (42.8-69.9%) | Reference |  |
| **PFS** |  |  |  | 0.4717 |
| A/A | 19/19 | 10.5 (2.8-39.0%) | 1.22 (0.71-2.11) |  |
| A/G or G/G | 50/53 | 22.6 (13.8-37.2%) | Reference |  |

Table S7: IRI and REGO PK parameters according to baseline characteristics

|  | **Concentration per visit**  **(ng/ml)** | **BMI** | | **Test**  **Wilcoxon**  **(p-value)** | **Previous gastrectomy** | | **Test**  **Wilcoxon**  **(p-value)** | **HER2 Status** | | **Test***  **(p-value)** |
| --- | --- | --- | --- | --- | --- | --- | --- | --- | --- | --- |
|  |  | **<25** | **≥ 25** |  | **NO** | **YES** |  | **HER2+** | **HER2-** |  |
| **IRI (ng/mL)** | | | | | | | | | | |
| **Cycle1 / Day1 Post Dose** | | | | | | | | | | |
| N / missing | 35 / 0 | 20 / 0 | 15 / 0 | 0.7138 | 26 / 0 | 9 / 0 | 0.0761 | 8 / 0 | 22 / 0 | 1.0000 |
| Mean  (Std) | 1405.6  (1411.8) | 1434.1  (1698.7) | 1367.8  (960.1) |  | 1492.0  (1490.7) | 1156.0  (1196.5) |  | 1101.2  (347.6) | 1376.7  (1649.6) |  |
| Median  (min; max) | 1089.7  (25.0; 8431.5) | 1056.0  (25.0; 8431.5) | 1318.3  (25.0; 4174.8) |  | 1220.9  (25.0; 8431.5) | 748.4  (25.0; 4174.8) |  | 1019.0  (571.6; 1715.9) | 1055.1  (25.0; 8431.5) |  |
| Q1-Q3 | 904.4-1507.9 | 843.5-1479.1 | 904.4-1543.4 |  | 953.2-1543.4 | 670.6-1093.1 |  | 942.5-1299.8 | 746.7-1450.2 |  |
| Cycle1 / Day15 |  |  |  |  |  |  |  |  |  |  |
| N / missing | 27 / 0 | 15 / 0 | 12 / 0 | 0.0231 | 20 / 0 | 7 / 0 | 0.2924 | 7 / 0 | 15 / 0 | 0.8878 |
| Mean  (Std) | 1258.9  (1194.4) | 869.6  (612.3) | 1745.4  (1558.8) |  | 1145.6  (659.0) | 1582.5  (2154.5) |  | 1007.8  (461.0) | 1006.2  (578.3) |  |
| Median  (min; max) | 1125.5  (25.0; 6394.8) | 1035.5  (25.0; 1973.6) | 1331.4  (307.0; 6394.8) |  | 1185.7  (25.0; 2490.1) | 981.9  (25.0; 6394.8) |  | 1083.3  (25.0; 1468.8) | 1088.6  (25.0; 1973.6) |  |
| Q1-Q3 | 761.0-1499.5 | 25.0-1254.2 | 1146.5-1678.7 |  | 899.0-1525.2 | 700.4-1125.5 |  | 981.9-1254.2 | 700.4-1499.5 |  |
| **Cycle2 / Day1 Post Dose** | | | | | | | | | | |
| N / missing | 26 / 0 | 15 / 0 | 11 / 0 | 0.8350 | 19 / 0 | 7 / 0 | 0.9076 | 7 / 0 | 14 / 0 | 0.7082 |
| Mean  (Std) | 1017.5  (808.8) | 1063.7  (1020.6) | 954.6  (412.0) |  | 926.3  (561.9) | 1265.2  (1296.4) |  | 1379.0  (1209.7) | 904.6  (648.5) |  |
| Median  (min; max) | 1102.7  (25.0; 3902.5) | 1101.0  (25.0; 3902.5) | 1104.4  (25.0; 1350.6) |  | 1104.4  (25.0; 1663.6) | 1101.0  (25.0; 3902.5) |  | 1210.3  (25.0; 3902.5) | 1144.6  (25.0; 1669.4) |  |
| Q1-Q3 | 358.7-1350.6 | 25.0-1517.1 | 901.7-1224.2 |  | 358.7-1350.6 | 150.0-1669.4 |  | 762.8-1431.4 | 150.0-1498.8 |  |
| **Cycle2 / Day15** | | | | | | | | | | |
| N / missing | 25 / 0 | 12 / 0 | 13 / 0 | 0.6053 | 18 / 0 | 7 / 0 | 0.7854 | 7 / 0 | 13 / 0 | **0.0476** |
| Mean  (Std) | 1340.4  (889.2) | 1135.6  (520.7) | 1529.5  (1118.9) |  | 1358.6  (977.2) | 1293.7  (673.3) |  | 1880.5  (1346.2) | 1010.0  (417.2) |  |
| Median  (min; max) | 1161.9  (25.0; 4783.6) | 1111.6  (25.0; 1960.3) | 1293.3  (538.2; 4783.6) |  | 1278.4  (25.0; 4783.6) | 1061.2  (798.7; 2725.1) |  | 1509.3  (852.1; 4783.6) | 1061.2  (25.0; 1503.4) |  |
| Q1-Q3 | 881.1-1503.4 | 840.2-1506.4 | 1022.8-1457.9 |  | 881.1-1509.3 | 852.1-1503.4 |  | 881.1-1960.3 | 798.7-1293.3 |  |
| **SN38 (ng/mL)** | | | | | | | | | | |
| **Cycle1 / Day1 Post Dose** | | | | | | | | | | |
| N / missing | 35 / 0 | 20 / 0 | 15 / 0 | 0.2301 | 26 / 0 | 9 / 0 | **0.0329** | 8 / 0 | 22 / 0 | 0.5735 |
| Mean  (Std) | 22.5  (14.7) | 21.7  (16.8) | 23.5  (11.9) |  | 24.5  (14.9) | 16.6  (13.1) |  | 23.8  (13.6) | 21.8  (15.8) |  |
| Median  (min; max) | 18.6  (2.5; 72.9) | 17.5  (2.5; 72.9) | 19.8  (2.5; 48.6) |  | 19.7  (2.5; 72.9) | 14.2  (2.5; 48.6) |  | 22.4  (11.1; 53.7) | 18.6  (2.5; 72.9) |  |
| Q1-Q3 | 14.2-26.0 | 12.7-24.4 | 15.7-34.8 |  | 16.2-27.6 | 11.1-18.1 |  | 13.5-26.8 | 12.9-24.4 |  |
| **Cycle1 / Day15** | | | | | | | | | | |
| N / missing | 27 / 0 | 15 / 0 | 12 / 0 | 0.0668 | 20 / 0 | 7 / 0 | 0.7817 | 7 / 0 | 15 / 0 | 0.7507 |
| Mean  (Std) | 19.5  (12.3) | 16.2  (12.7) | 23.7  (10.8) |  | 19.6  (11.7) | 19.4  (14.9) |  | 19.7  (13.4) | 18.1  (10.8) |  |
| Median  (min; max) | 16.9  (2.5; 48.8) | 16.2  (2.5; 46.0) | 20.2  (13.9; 48.8) |  | 17.5  (2.5; 46.0) | 16.2  (2.5; 48.8) |  | 16.6  (2.5; 46.0) | 15.2  (2.5; 39.2) |  |
| Q1-Q3 | 13.9-25.6 | 2.5-18.0 | 14.8-27.2 |  | 14.1-25.4 | 9.6-26.9 |  | 14.3-25.2 | 13.8-25.6 |  |
| **Cycle2 / Day1 Post Dose** | | | | | | | | | | |
| N / missing | 26 / 0 | 15 / 0 | 11 / 0 | 0.6571 | 19 / 0 | 7 / 0 | 0.9074 | 7 / 0 | 14 / 0 | 0.5477 |
| Mean  (Std) | 17.4  (10.6) | 16.5  (12.6) | 18.6  (7.4) |  | 17.9  (10.8) | 16.3  (10.6) |  | 20.0  (10.8) | 15.9  (11.1) |  |
| Median  (min; max) | 16.3  (2.5; 35.8) | 14.6  (2.5; 35.8) | 21.5  (2.5; 28.3) |  | 15.9  (2.5; 35.8) | 16.6  (2.5; 28.3) |  | 21.5  (2.5; 33.5) | 15.3  (2.5; 35.8) |  |
| Q1-Q3 | 13.2-24.7 | 2.5-29.6 | 14.0-24.7 |  | 13.2-24.7 | 2.5-25.9 |  | 13.2-29.6 | 2.5-24.7 |  |
| **Cycle2 / Day15** | | | | | | | | | | |
| N / missing | 25 / 0 | 12 / 0 | 13 / 0 | 0.3143 | 18 / 0 | 7 / 0 | 0.4139 | 7 / 0 | 13 / 0 | 0.5791 |
| Mean  (Std) | 26.1  (14.9) | 24.6  (17.6) | 27.5  (12.4) |  | 26.8  (15.1) | 24.3  (15.2) |  | 30.7  (18.6) | 24.1  (14.4) |  |
| Median  (min; max) | 22.2  (2.5; 63.7) | 18.9  (2.5; 63.7) | 22.2  (14.2; 53.4) |  | 23.6  (2.5; 63.7) | 17.4  (10.7; 48.6) |  | 30.7  (10.7; 63.7) | 20.6  (2.5; 53.4) |  |
| Q1-Q3 | 14.5-34.5 | 12.9-32.6 | 18.1-36.3 |  | 17.6-34.5 | 13.3-42.7 |  | 14.5-42.9 | 14.2-27.3 |  |
| **Ratio SN38/CPT11** | | | | | | | | | | |
| **Cycle1 / Day1 Post Dose** | | | | | | | | | | |
| N / missing | 35 / 0 | 20 / 0 | 15 / 0 | 0.3337 | 26 / 0 | 9 / 0 | 0.8208 | 8 / 0 | 22 / 0 | 0.5266 |
| Mean  (Std) | 0.023  (0.021) | 0.021  (0.020) | 0.025  (0.022) |  | 0.022  (0.018) | 0.025  (0.028) |  | 0.021  (0.007) | 0.026  (0.025) |  |
| Median  (min; max) | 0.017  (0.008; 0.100) | 0.015  (0.009; 0.100) | 0.018  (0.008; 0.100) |  | 0.017  (0.008; 0.100) | 0.017  (0.011; 0.100) |  | 0.021  (0.012; 0.031) | 0.017  (0.009; 0.100) |  |
| Q1-Q3 | 0.012-0.024 | 0.011-0.023 | 0.015-0.024 |  | 0.012-0.024 | 0.013-0.019 |  | 0.015-0.026 | 0.012-0.024 |  |
| **Cycle1 / Day15** | | | | | | | | | | |
| N / missing | 27 / 0 | 15 / 0 | 12 / 0 | 0.1122 | 20 / 0 | 7 / 0 | 0.9118 | 7 / 0 | 15 / 0 | 0.9437 |
| Mean  (Std) | 0.031  (0.031) | 0.041  (0.038) | 0.019  (0.012) |  | 0.032  (0.032) | 0.028  (0.032) |  | 0.031  (0.032) | 0.031  (0.030) |  |
| Median  (min; max) | 0.016  (0.008; 0.100) | 0.016  (0.009; 0.100) | 0.015  (0.008; 0.050) |  | 0.016  (0.008; 0.100) | 0.016  (0.008; 0.100) |  | 0.016  (0.012; 0.100) | 0.018  (0.009; 0.100) |  |
| Q1-Q3 | 0.013-0.037 | 0.014-0.100 | 0.012-0.021 |  | 0.013-0.040 | 0.014-0.024 |  | 0.014-0.043 | 0.013-0.037 |  |
| **Cycle2 / Day1 Post Dose** | | | | | | | | | | |
| N / missing | 26 / 0 | 15 / 0 | 11 / 0 | 0.9170 | 19 / 0 | 7 / 0 | 0.3241 | 7 / 0 | 14 / 0 | 0.7646 |
| Mean  (Std) | 0.035  (0.033) | 0.039  (0.038) | 0.028  (0.025) |  | 0.037  (0.034) | 0.028  (0.032) |  | 0.029  (0.032) | 0.036  (0.035) |  |
| Median  (min; max) | 0.020  (0.007; 0.100) | 0.022  (0.007; 0.100) | 0.020  (0.010; 0.100) |  | 0.020  (0.009; 0.100) | 0.017  (0.007; 0.100) |  | 0.018  (0.007; 0.100) | 0.019  (0.009; 0.100) |  |
| Q1-Q3 | 0.015-0.031 | 0.012-0.100 | 0.018-0.026 |  | 0.017-0.037 | 0.010-0.026 |  | 0.010-0.031 | 0.013-0.037 |  |
| **Cycle2 / Day15** | | | | | | | | | | |
| N / missing | 25 / 0 | 12 / 0 | 13 / 0 | 0.6833 | 18 / 0 | 7 / 0 | 0.1228 | 7 / 0 | 13 / 0 | 0.1538 |
| Mean  (Std) | 0.025  (0.019) | 0.028  (0.027) | 0.021  (0.009) |  | 0.026  (0.020) | 0.021  (0.018) |  | 0.018  (0.009) | 0.031  (0.025) |  |
| Median  (min; max) | 0.018  (0.009; 0.100) | 0.016  (0.011; 0.100) | 0.019  (0.009; 0.038) |  | 0.022  (0.009; 0.100) | 0.015  (0.011; 0.061) |  | 0.016  (0.009; 0.036) | 0.022  (0.011; 0.100) |  |
| Q1-Q3 | 0.015-0.024 | 0.014-0.029 | 0.016-0.024 |  | 0.016-0.028 | 0.013-0.018 |  | 0.013-0.023 | 0.015-0.037 |  |
| **Regorafenib (ng/mL)** | | | | | | | | | | |
| **Cycle1 / Day8** | | | | | | | | | | |
| N / missing | 28 / 0 | 14 / 0 | 14 / 0 | 0.2413 | 21 / 0 | 7 / 0 | 0.4904 | 6 / 0 | 18 / 0 | 0.6171 |
| Mean  (Std) | 2399.2  (1668.0) | 2842.3  (2014.6) | 1956.0  (1138.9) |  | 2345.8  (1182.4) | 2559.1  (2796.6) |  | 2763.1  (1648.0) | 2413.0  (1751.3) |  |
| Median  (min; max) | 2058.3  (235.7; 8634.0) | 2137.7  (918.6; 8634.0) | 1859.9  (235.7; 3912.2) |  | 2063.7  (783.3; 5574.8) | 1980.0  (235.7; 8634.0) |  | 2419.1  (918.6; 5574.8) | 2058.3  (783.3; 8634.0) |  |
| Q1-Q3 | 1397.3-2952.6 | 1794.7-2958.4 | 988.0-2946.8 |  | 1667.0-2958.4 | 918.6-2596.8 |  | 1685.3-3561.4 | 1445.8-2946.8 |  |
| **Cycle2 / Day8** | | | | | | | | | | |
| N / missing | 20 / 0 | 9 / 0 | 11 / 0 | 0.6485 | 16 / 0 | 4 / 0 | 0.0982 | 5 / 0 | 12 / 0 | 0.1023 |
| Mean  (Std) | 1964.0  (1218.7) | 1946.5  (1499.9) | 1978.3  (1010.8) |  | 1773.4  (1215.9) | 2726.2  (1023.0) |  | 2772.1  (1651.5) | 1545.6  (935.5) |  |
| Median  (min; max) | 1672.8  (262.5; 4651.9) | 1841.3  (262.5; 4651.9) | 1504.2  (871.8; 3382.7) |  | 1283.4  (262.5; 4651.9) | 2624.3  (1841.3; 3814.9) |  | 3311.6  (990.1; 4651.9) | 1324.8  (262.5; 3047.6) |  |
| Q1-Q3 | 964.8-3012.4 | 857.8-2621.3 | 1092.1-3047.6 |  | 905.6-2799.3 | 1853.6-3598.8 |  | 1092.1-3814.9 | 864.8-2243.6 |  |
| **M2 (ng/mL)** | | | | | | | | | | |
| **Cycle1 / Day8** | | | | | | | | | | |
| N / missing | 28 / 0 | 14 / 0 | 14 / 0 | 0.0565 | 21 / 0 | 7 / 0 | 0.9155 | 6 / 0 | 18 / 0 | 0.7642 |
| Mean  (Std) | 1341.7  (970.2) | 1680.0  (970.5) | 1003.3  (875.6) |  | 1284.4  (843.3) | 1513.4  (1348.9) |  | 1340.4  (959.2) | 1482.4  (1030.9) |  |
| Median  (min; max) | 1095.4  (100.0; 3964.9) | 1593.4  (390.3; 3964.9) | 735.2  (100.0; 2990.7) |  | 958.1  (227.5; 2990.7) | 1232.6  (100.0; 3964.9) |  | 1180.9  (390.3; 2724.6) | 1349.5  (227.5; 3964.9) |  |
| Q1-Q3 | 511.2-2063.3 | 955.8-2079.7 | 494.0-1276.4 |  | 515.5-2054.7 | 506.8-2581.8 |  | 494.0-2071.9 | 670.8-2079.7 |  |
| **Cycle2 / Day8** | | | | | | | | | | |
| N / missing | 20 / 0 | 9 / 0 | 11 / 0 | 0.8194 | 16 / 0 | 4 / 0 | 0.1424 | 5 / 0 | 12 / 0 | 0.4922 |
| Mean  (Std) | 1139.3  (829.3) | 1212.4  (981.0) | 1079.5  (726.7) |  | 1009.1  (821.2) | 1660.1  (729.3) |  | 1514.4  (1277.9) | 1028.2  (688.4) |  |
| Median  (min; max) | 1019.3  (100.0; 2712.7) | 1069.2  (100.0; 2712.7) | 920.7  (100.0; 2267.7) |  | 899.3  (100.0; 2712.7) | 1469.8  (1007.3; 2693.5) |  | 1820.7  (100.0; 2712.7) | 964.0  (100.0; 2267.7) |  |
| Q1-Q3 | 475.4-1705.5 | 455.2-1590.3 | 495.5-1820.7 |  | 350.1-1502.2 | 1178.3-2141.9 |  | 244.9-2693.5 | 566.4-1387.0 |  |
| **M5 (ng/mL)** | | | | | | | | | | |
| **Cycle1 / Day8** | | | | | | | | | | |
| N / missing | 28 / 0 | 14 / 0 | 14 / 0 | 0.2234 | 21 / 0 | 7 / 0 | 0.6712 | 6 / 0 | 18 / 0 | 0.7642 |
| Mean  (Std) | 2383.3  (1581.6) | 2805.1  (1880.9) | 1961.5  (1128.8) |  | 2356.1  (1184.2) | 2465.1  (2563.4) |  | 2734.3  (1704.0) | 2397.8  (1603.1) |  |
| Median  (min; max) | 2080.0  (261.9; 7987.7) | 2162.9  (876.7; 7987.7) | 1849.6  (261.9; 3932.5) |  | 2018.5  (825.6; 5687.1) | 2154.1  (261.9; 7987.7) |  | 2217.3  (876.7; 5687.1) | 2147.8  (825.6; 7987.7) |  |
| Q1-Q3 | 1337.3-3051.2 | 1929.9-3219.5 | 1036.7-3005.1 |  | 1680.6-3097.3 | 876.7-2495.8 |  | 1779.7-3627.7 | 1382.8-3005.1 |  |
| **Cycle2 / Day8** | | | | | | | | | | |
| N / missing | 20 / 0 | 9 / 0 | 11 / 0 | 0.6485 | 16 / 0 | 4 / 0 | 0.0982 | 5 / 0 | 12 / 0 | 0.1264 |
| Mean  (Std) | 1993.1  (1227.2) | 1987.5  (1527.1) | 1997.7  (997.8) |  | 1801.5  (1216.0) | 2759.5  (1079.5) |  | 2781.8  (1650.1) | 1570.1  (959.4) |  |
| Median  (min; max) | 1670.2  (249.6; 4738.2) | 1783.1  (249.6; 4738.2) | 1557.2  (876.0; 3433.8) |  | 1347.6  (249.6; 4738.2) | 2666.9  (1783.1; 3921.1) |  | 3058.9  (1093.0; 4738.2) | 1371.2  (249.6; 3127.5) |  |
| Q1-Q3 | 1000.3-3055.9 | 857.4-2673.9 | 1097.6-3058.9 |  | 891.8-2863.4 | 1841.5-3677.5 |  | 1097.6-3921.1 | 866.7-2286.9 |  |
| **Regorafenib+M2+M5** | | | | | | | | | | |
| **Cycle1 / Day8** | | | | | | | | | | |
| N / missing | 28 / 0 | 14 / 0 | 14 / 0 | 0.1478 | 21 / 0 | 7 / 0 | 0.7103 | 6 / 0 | 18 / 0 | 0.8155 |
| Mean  (Std) | 6124.1  (4035.0) | 7327.5  (4737.6) | 4920.8  (2872.3) |  | 5986.3  (2966.2) | 6537.6  (6608.3) |  | 6837.8  (4178.8) | 6293.2  (4200.0) |  |
| Median  (min; max) | 5718.6  (597.6; 20586.6) | 5876.7  (2486.6; 20586.6) | 4752.9  (597.6; 9261.0) |  | 5734.3  (1842.0; 13986.5) | 5400.5  (597.6; 20586.6) |  | 5718.6  (2486.6; 13986.5) | 5876.7  (1842.0; 20586.6) |  |
| Q1-Q3 | 3465.9-7399.9 | 4385.5-7864.2 | 2435.6-6877.7 |  | 3855.3-7864.2 | 2486.6-6935.5 |  | 3855.3-9261.0 | 3784.4-6935.5 |  |
| **Cycle2 / Day8** | | | | | | | | | | |
| N / missing | 20 / 0 | 9 / 0 | 11 / 0 | 0.8197 | 16 / 0 | 4 / 0 | 0.1430 | 5 / 0 | 12 / 0 | 0.2684 |
| Mean  (Std) | 5096.4  (3193.4) | 5146.4  (3942.9) | 5055.5  (2633.4) |  | 4584.1  (3177.1) | 7145.8  (2667.5) |  | 7068.2  (4561.8) | 4143.9  (2510.9) |  |
| Median  (min; max) | 4297.3  (967.3; 12102.8) | 4631.7  (967.3; 12102.8) | 3962.8  (1915.4; 8372.4) |  | 3473.7  (967.3; 12102.8) | 6761.0  (4631.7; 10429.5) |  | 8191.2  (2183.1; 12102.8) | 3473.7  (967.3; 8372.4) |  |
| Q1-Q3 | 2567.3-8177.8 | 2183.1-6478.8 | 2700.0-8189.8 |  | 2308.9-7334.3 | 4993.9-9297.7 |  | 2434.6-10429.5 | 2307.7-5917.5 |  |

*Wilcoxon

Table S8: IRI and REGO PK parameters according to AEs

|  | **At least one adverse event of grade≥3** | | **Test**  **Wilcoxon**  **(p-value)** | **Grade 5 adverse event** | | **Test**  **Wilcoxon**  **(p-value)** |
| --- | --- | --- | --- | --- | --- | --- |
|  | **No N=7** | **Yes N=28** |  | **No (N=29)** | **Yes (N=6)** |  |
| **IRI (ng/mL)** | | | | | | |
| **Cycle1 / Day1 Post Dose** | | | | | | |
| N / missing | 7 / 0 | 28 / 0 | 0.4455 | 29 / 0 | 6 / 0 | 0.0216 |
| Mean (Std) | 1130 (626) | 1474 (1549) |  | 1157 (764) | 2607 (2863) |  |
| Median (min; max) | 946 (450; 2351) | 1122 (25; 8432) |  | 1016 (25; 4175) | 1558 (1090; 8432) |  |
| Q1-Q3 | 730-1508 | 940-1497 |  | 748-1382 | 1292-1716 |  |
| **Cycle1 / Day15** | | | | | | |
| N / missing | 7 / 0 | 20 / 0 | 0.7817 | 24 / 0 | 3 / 0 | 0.1643 |
| Mean (Std) | 1067 (822) | 1326 (1312) |  | 1327 (1240) | 712 (595) |  |
| Median (min; max) | 1083 (25; 2490) | 1147 (25; 6395) |  | 1172 (25; 6395) | 1036 (25; 1074) |  |
| Q1-Q3 | 307-1551 | 872-1484 |  | 762-1525 | 25-1074 |  |
| **Cycle2 / Day1 Post Dose** | | | | | | |
| N / missing | 7 / 0 | 19 / 0 | 0.1173 | 23 / 0 | 3 / 0 | 0.2270 |
| Mean (Std) | 662 (506) | 1149 (869) |  | 977 (848) | 1329 (310) |  |
| Median (min; max) | 763 (25; 1431) | 1188 (25; 3903) |  | 1101 (25; 3903) | 1499 (970; 1517) |  |
| Q1-Q3 | 150-1000 | 902-1499 |  | 150-1315 | 970-1517 |  |
| **Cycle2 / Day15** | | | | | | |
| N / missing | 7 / 0 | 18 / 0 | 0.1932 | 23 / 0 | 2 / 0 | 0.8023 |
| Mean (Std) | 1038 (412) | 1458 (1002) |  | 1378 (879) | 904 (1243) |  |
| Median (min; max) | 881 (538; 1688) | 1278 (25; 4784) |  | 1162 (538; 4784) | 904 (25; 1782) |  |
| Q1-Q3 | 799-1509 | 1023-1503 |  | 881-1503 | 25-1782 |  |
| **SN38 (ng/mL)** | | | | | | |
| **Cycle1 / Day1 Post Dose** | | | | | | |
| N / missing | 7 / 0 | 28 / 0 | 0.5918 | 29 / 0 | 6 / 0 | 0.0919 |
| Mean (Std) | 18 (4) | 24 (16) |  | 20 (11) | 37 (24) |  |
| Median (min; max) | 19 (13; 23) | 19 (3; 73) |  | 18 (3; 49) | 30 (13; 73) |  |
| Q1-Q3 | 14-20 | 14-31 |  | 14-23 | 19-54 |  |
| **Cycle1 / Day15** | | | | | | |
| N / missing | 7 / 0 | 20 / 0 | 1.0000 | 24 / 0 | 3 / 0 | 0.4396 |
| Mean (Std) | 17 (7) | 21 (14) |  | 18 (11) | 29 (23) |  |
| Median (min; max) | 17 (3; 28) | 17 (3; 49) |  | 17 (3; 49) | 38 (3; 46) |  |
| Q1-Q3 | 15-20 | 14-26 |  | 14-23 | 16862 |  |
| **Cycle2 / Day1 Post Dose** | | | | | | |
| N / missing | 7 / 0 | 19 / 0 | 0.1905 | 23 / 0 | 3 / 0 | 0.0825 |
| Mean (Std) | 14 (10) | 19 (11) |  | 16 (10) | 27 (8) |  |
| Median (min; max) | 13 (3; 34) | 22 (3; 36) |  | 15 (3; 36) | 30 (19; 34) |  |
| Q1-Q3 | 42430 | 14-26 |  | 45717 | 19-34 |  |
| **Cycle2 / Day15** | | | | | | |
| N / missing | 7 / 0 | 18 / 0 | 0.8797 | 23 / 0 | 2 / 0 | 1.0000 |
| Mean (Std) | 26 (13) | 26 (16) |  | 26 (12) | 33 (43) |  |
| Median (min; max) | 22 (13; 49) | 21 (3; 64) |  | 22 (11; 53) | 33 (3; 64) |  |
| Q1-Q3 | 15-35 | 14-36 |  | 15-35 | 23437 |  |
| **Ratio SN38/CPT11** | | | | | | |
| **Cycle1 / Day1 Post Dose** | | | | | | |
| N / missing | 7 / 0 | 28 / 0 | 0.8528 | 29 / 0 | 6 / 0 | 0.5256 |
| Mean (Std) | 0.020 (0.012) | 0.024 (0.023) |  | 0.024 (0.022) | 0.018 (0.011) |  |
| Median (min; max) | 0.019 (0.008; 0.044) | 0.017 (0.009; 0.100) |  | 0.018 (0.008; 0.100) | 0.013 (0.009; 0.032) |  |
| Q1-Q3 | 0.011-0.024 | 0.012-0.023 |  | 0.012-0.023 | 0.012-0.031 |  |
| Cycle1 / Day15 |  |  | Wilcoxon |  |  | Wilcoxon |
| N / missing | 7 / 0 | 20 / 0 | 0.6180 | 24 / 0 | 3 / 0 | 0.0445 |
| Mean (Std) | 0.032 (0.033) | 0.030 (0.031) |  | 0.027 (0.029) | 0.060 (0.035) |  |
| Median (min; max) | 0.018 (0.008; 0.100) | 0.015 (0.008; 0.100) |  | 0.015 (0.008; 0.100) | 0.043 (0.037; 0.100) |  |
| Q1-Q3 | 0.014-0.050 | 0.013-0.035 |  | 0.013-0.023 | 0.037-0.100 |  |
| Cycle2 / Day1 Post Dose |  |  | Wilcoxon |  |  | Wilcoxon |
| N / missing | 7 / 0 | 19 / 0 | 1.0000 | 23 / 0 | 3 / 0 | 1.0000 |
| Mean (Std) | 0.032 (0.031) | 0.035 (0.035) |  | 0.036 (0.035) | 0.022 (0.009) |  |
| Median (min; max) | 0.018 (0.015; 0.100) | 0.020 (0.007; 0.100) |  | 0.020 (0.007; 0.100) | 0.022 (0.012; 0.031) |  |
| Q1-Q3 | 0.017-0.037 | 0.012-0.031 |  | 0.015-0.037 | 0.012-0.031 |  |
| Cycle2 / Day15 |  |  | Wilcoxon |  |  | Wilcoxon |
| N / missing | 7 / 0 | 18 / 0 | 0.2379 | 23 / 0 | 2 / 0 | 0.0508 |
| Mean (Std) | 0.027 (0.017) | 0.024 (0.021) |  | 0.021 (0.011) | 0.068 (0.045) |  |
| Median (min; max) | 0.022 (0.015; 0.061) | 0.017 (0.009; 0.100) |  | 0.017 (0.009; 0.061) | 0.068 (0.036; 0.100) |  |
| Q1-Q3 | 0.016-0.038 | 0.013-0.024 |  | 0.014-0.023 | 0.036-0.100 |  |
| **Regorafenib (ng/mL)** | | | | | | |
| **Cycle1 / Day8** | | | | | | |
| N / missing | 6 / 0 | 22 / 0 | 0.4840 | 23 / 0 | 5 / 0 | 0.1677 |
| Mean (Std) | 2678 (2948) | 2323 (1216) |  | 2270 (1707) | 2992 (1491) |  |
| Median (min; max) | 1676 (988; 8634) | 2132 (236; 5575) |  | 1980 (236; 8634) | 2279 (1950; 5575) |  |
| Q1-Q3 | 1015-2076 | 1446-2958 |  | 1015-2947 | 2200-2958 |  |
| Cycle2 / Day8 |  |  | Wilcoxon |  |  | Wilcoxon |
| N / missing | 3 / 0 | 17 / 0 | 0.5254 | 18 / 0 | 2 / 0 | 0.9498 |
| Mean (Std) | 1305 (276) | 2080 (1288) |  | 1890 (1059) | 2632 (2856) |  |
| Median (min; max) | 1422 (990; 1504) | 1866 (263; 4652) |  | 1673 (263; 3815) | 2632 (613; 4652) |  |
| Q1-Q3 | 990-1504 | 939-3048 |  | 990-2977 | 613-4652 |  |
| **M2 (ng/mL)** | | | | | | |
| **Cycle1 / Day8** | | | | | | |
| N / missing | 6 / 0 | 22 / 0 | 0.6341 | 23 / 0 | 5 / 0 | 0.2078 |
| Mean (Std) | 1367 (1385) | 1335 (868) |  | 1244 (975) | 1793 (904) |  |
| Median (min; max) | 974 (228; 3965) | 1095 (100; 2991) |  | 956 (100; 3965) | 1686 (505; 2725) |  |
| Q1-Q3 | 390-1670 | 516-2072 |  | 507-2055 | 1466-2582 |  |
| Cycle2 / Day8 |  |  | Wilcoxon |  |  | Wilcoxon |
| N / missing | 3 / 0 | 17 / 0 | 0.2891 | 18 / 0 | 2 / 0 | 0.9497 |
| Mean (Std) | 670 (499) | 1222 (858) |  | 1110 (747) | 1406 (1847) |  |
| Median (min; max) | 878 (100; 1031) | 1069 (100; 2713) |  | 1019 (100; 2694) | 1406 (100; 2713) |  |
| Q1-Q3 | 100-1031 | 496-1821 |  | 496-1590 | 100-2713 |  |
| **M5 (ng/mL)** | | | | | | |
| **Cycle1 / Day8** | | | | | | |
| N / missing | 6 / 0 | 22 / 0 | 0.3855 | 23 / 0 | 5 / 0 | 0.2078 |
| Mean (Std) | 2604 (2663) | 2323 (1229) |  | 2242 (1583) | 3032 (1566) |  |
| Median (min; max) | 1730 (1037; 7988) | 2163 (262; 5687) |  | 1988 (262; 7988) | 2172 (1930; 5687) |  |
| Q1-Q3 | 1153-1988 | 1383-3097 |  | 1153-3005 | 2154-3220 |  |
| Cycle2 / Day8 |  |  | Wilcoxon |  |  | Wilcoxon |
| N / missing | 3 / 0 | 17 / 0 | 0.5254 | 18 / 0 | 2 / 0 | 0.9498 |
| Mean (Std) | 1387 (255) | 2100 (1303) |  | 1914 (1063) | 2705 (2876) |  |
| Median (min; max) | 1510 (1093; 1557) | 1900 (250; 4738) |  | 1670 (250; 3921) | 2705 (671; 4738) |  |
| Q1-Q3 | 1093-1557 | 908-3059 |  | 1093-3053 | 671-4738 |  |
| **Regorafenib+M2+M5** | | | | | | |
| **Cycle1 / Day8** | | | | | | |
| N / missing | 6 / 0 | 22 / 0 | 0.4169 | 23 / 0 | 5 / 0 | 0.1500 |
| Mean (Std) | 6649 (6940) | 5981 (3060) |  | 5756 (4089) | 7818 (3682) |  |
| Median (min; max) | 4240 (2252; 20587) | 5877 (598; 13987) |  | 5401 (598; 20587) | 6936 (4386; 13987) |  |
| Q1-Q3 | 2839-5734 | 3784-7864 |  | 2839-6878 | 5917-7864 |  |
| Cycle2 / Day8 |  |  | Wilcoxon |  |  | Wilcoxon |
| N / missing | 3 / 0 | 17 / 0 | 0.3971 | 18 / 0 | 2 / 0 | 0.9498 |
| Mean (Std) | 3362 (1021) | 5403 (3364) |  | 4913 (2768) | 6743 (7580) |  |
| Median (min; max) | 3939 (2183; 3963) | 4732 (967; 12103) |  | 4297 (967; 10430) | 6743 (1384; 12103) |  |
| Q1-Q3 | 2183-3963 | 2700-8190 |  | 2700-8166 | 1384-12103 |  |

Figure S6: Kaplan-Meier estimates of OS_REGO_ and PFS_REGO_

1. **OS_REGO_**


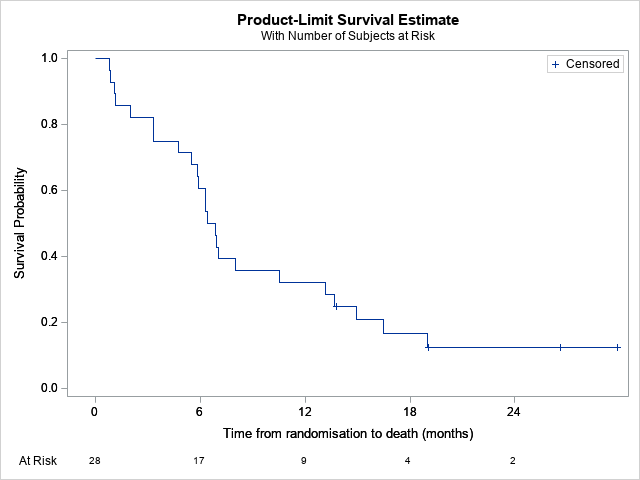


1. **PFS_REGO_**


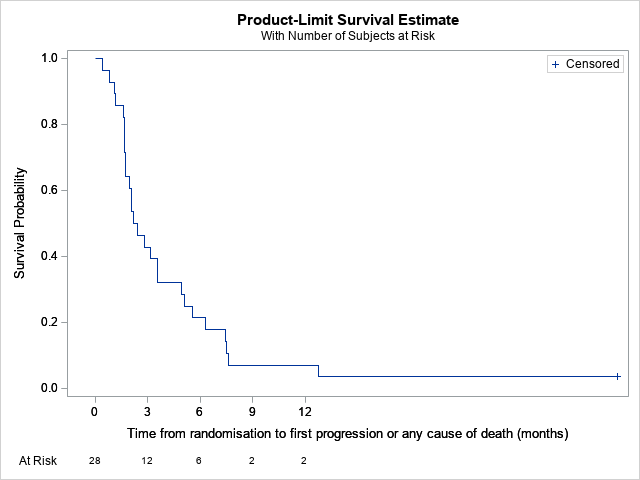


The REGO parameters population included the 28 patients randomized in the REGIRI arm who had at least one blood sample collected at C1D8.

A. OS_REGO_ was defined as the time from C1D8 until death of any cause. If a patient was alive at the database cut-off date, then the patient was censored at the last date of follow-up.

B. PFS_REGO_ was defined as the time from C1D8 to disease progression (radiological or clinical) or death of any cause, whichever occured first. Patients without tumour progression or death at the time of analysis were censored at the date of their last tumour assessment.

Table S9: Univariate analysis for prognostic factors of OS_REGO_ and PFS_REGO_ at C1D8

|  | **Event/Total** | **Unstratified Hazard Ratio** | **p-value^#^** |
| --- | --- | --- | --- |
|  |  | **(95% CI)**^Cox^ |  |
| **OS** | 24/28 |  |  |
| Regorafenib (/100) |  | 1.00 (0.98-1.02) | 0.8312 |
| M-2 (/100) |  | 1.02 (0.99-1.06) | 0.2808 |
| M-5 (/100) |  | 1.00 (0.98-1.02) | 0.8594 |
| Regorafenib+M-2+M-5 (/100) |  | 1.00 (0.99-1.01) | 0.9258 |
| Baseline characteristics |  |  |  |
| BMI | 24/28 | 0.93 (0.84-1.03) | 0.1678 |
| Previous gastrectomy |  |  | 0.8534 |
| No | 18/21 | Reference |  |
| Yes | 6/7 | 1.09 (0.43-2.79) |  |
| HER2 Status |  |  | 0.7801 |
| HER2+ | 6/6 | 0.87 (0.34-2.27) |  |
| HER2- | 15/18 | Reference |  |
| **PFS** | 27/28 |  |  |
| Regorafenib (/100) |  | 1.00 (0.97-1.02) | 0.7041 |
| M-2 (/100) |  | 1.03 (0.99-1.07) | 0.1289 |
| M-5 (/100) |  | 0.99 (0.97-1.02) | 0.6730 |
| Regorafenib+M2+M5 (/100) |  | 1.00 (0.99-1.01) | 0.9535 |
| Baseline characteristics |  |  |  |
| BMI | 27/28 | 0.86 (0.77-0.96) | **0.0065** |
| Previous gastrectomy |  |  | **0.0258** |
| No | 20/21 | Reference |  |
| Yes | 7/7 | 3.23 (1.22-8.55) |  |
| HER2 Status |  |  | 0.2887 |
| HER2+ | 6/6 | 0.58 (0.22-1.58) |  |
| HER2- | 18/18 | Reference |  |

^Cox^Cox model; ^#^Likelihood-ratio test

Table S10: Number of administered cycles per subgroups

|  | **REGIRI (N=36)** | | | **IRI (N=36)** | | | **All** |
| --- | --- | --- | --- | --- | --- | --- | --- |
|  | **UGT1A1*1** | **UGT1A1 *28** | **UGT1A1*1*28** | **UGT1A1*1** | **UGT1A1 *28** | **UGT1A1*1*28** |  |
|  | **N=14** | **N=4** | **N=18** | **N=17** | **N=6** | **N=13** | **N=72** |
| N / missing | 14 / 0 | 4 / 0 | 18 / 0 | 17 / 0 | 6 / 0 | 13 / 0 | 72 / 0 |
| Mean (Std) | 5.14 (4.99) | 2.50 (1.29) | 3.56 (2.12) | 3.53 (2.43) | 3.33 (1.51) | 3.31 (2.75) | 3.74 (3.00) |
| Median (min; max) | 3.00 (1.00; 18.00) | 2.50 (1.00; 4.00) | 3.00 (1.00; 8.00) | 2.00 (1.00; 9.00) | 3.00 (2.00; 6.00) | 2.00 (1.00; 10.00) | 3.00 (1.00; 18.00) |
| Q1-Q3 | 2.00-6.00 | 1.50-3.50 | 2.00-4.00 | 2.00-4.00 | 2.00-4.00 | 2.00-3.00 | 2.00-4.00 |
